# Obesity and Suicide Behavior in Young Latina Adults of Mexican Heritage: The Mediating Influences of Sleep Problems, Health Problems, and Dissatisfaction with Personal Appearance

**DOI:** 10.3390/bs16030442

**Published:** 2026-03-17

**Authors:** Joseph D. Hovey, Liza Talavera-Garza, Glenn Gray, Isabella A. Cruz, Nadeen Salhadar, Monica E. Ochoa

**Affiliations:** 1 Department of Psychological Science, The University of Texas Rio Grande Valley, Edinburg, TX 78539, USA; 2 Student Health Services, The University of Texas Rio Grande Valley, Edinburg, TX 78539, USA; 3 Department of Psychology, The University of Texas at San Antonio, San Antonio, TX 78249, USA

**Keywords:** obesity, suicidal ideation, suicide attempts, depression, sleep, insomnia, physical health, personal appearance, body mass index, Latina adults

## Abstract

Although research findings have indicated that obesity is associated with suicide behavior, especially in females, scant research has examined mediators in this relationship. Moreover, although suicides have increased in young Latina adults in the United States, no published studies have examined obesity and suicide risk in young Latina adults. The present study thus examined the relationship of obesity to suicide behavior in young Latina adults and assessed sleep problems, health problems, and dissatisfaction with personal appearance as possible mediators. Participants were 401 female students of Mexican heritage from South Texas who completed the National College Health Assessment II, from which data on suicide behavior, depression, sleep and health problems, personal appearance satisfaction, and body mass index were obtained. Obesity was significantly associated with sleep problems, health problems, dissatisfaction with personal appearance, depression, suicidal ideation, and suicide attempts. Parallel multiple mediation analyses and Sobel tests indicate that obesity had significant indirect effects on suicidal ideation through sleep problems, health problems, and personal appearance dissatisfaction. The present findings strongly suggest that sleep problems, health problems, and personal appearance dissatisfaction mediate the relationship between obesity and suicide behavior and thus help explain how obesity and suicide behavior are connected. Finally, the present study identifies suicide risk factors pertinent to young Latina adults while also identifying possible pathways for the prevention and treatment of suicide.

## 1. Introduction

An emerging body of research has shown that obesity is related to suicide behavior. For example, obesity has been associated with suicide attempts in male and female adolescents (Swahn et al., 2009) and adults (Dong et al., 2006). More common are findings, however, that indicated the associations of obesity to suicidal ideation and suicide attempts in adolescents (M. Zhang et al., 2022) and adults (Branco et al., 2017; Carpenter et al., 2000; Wagner et al., 2013; J. Zhang et al., 2013) were specific to females.

Because it is unclear exactly *why* obesity is associated with suicide behavior, authors (Klinitzke et al., 2013; Mukamal et al., 2009) have argued for research that examines mediators that would help explain the connection between obesity and suicide behavior. Scant research has examined possible mediators, however. Such research would not only help move this area of research forward but also provide possible pathways for intervention and prevention efforts.

### 1.1. Suicide Behavior in Latina College Students

Although the suicide rate in young Latino/a adults in the United States has increased by 39% during the past decade (Saunders & Panchal, 2023), scant research has focused on suicide behavior in young Latina adults. For example, Chesin and Jeglic (2012) examined data from 554 Latina undergraduate students and found that more than one in seven of the students had made a past suicide attempt, one in five reported current suicidal ideation, and one in nine reported thoughts of engaging in future suicide behavior. In addition, they found that suicide behavior among Latinas was associated with loneliness, hopelessness, depression, and a less positive social problem-solving orientation.

In a sample of 156 Latino/a undergraduate students (78% of whom were female), Chang et al. (2019) examined the influence of psychological vulnerabilities on suicide risk. They found that loneliness and depression were significant predictors of hopelessness and suicide behavior. In addition, they found that the interaction between loneliness and depression significantly predicted both hopelessness and suicide behavior. Their findings suggest that loneliness and depression are unique predictors of suicide behavior in Latinas and that Latinas who are depressed and socially isolated demonstrate greater suicide risk than Latinas who are depressed but not socially isolated.

To note, the current literature on young Latina adult suicide risk is limited to the examination of the above psychological vulnerabilities as predictors of suicide. No published research has examined obesity and suicide behavior in young Latina adults.

### 1.2. Purposes and Hypotheses

As mentioned, although research has found that obesity is associated with suicide behavior, especially among women, little to no studies have examined possible mediators in this relationship. Moreover, although suicides have increased in young Latina adults, few studies have assessed suicide thoughts and behavior in Latina college students. The purposes of the present study were thus to examine the relationship of obesity to suicidal ideation in young Latina college students and to assess possible mediators that may help explain this relationship.

We expected that obesity would be significantly associated with sleep problems, health problems, dissatisfaction with personal appearance, depression, suicidal ideation, and suicide attempts. We also expected that sleep problems, health problems, and dissatisfaction with personal appearance would be significantly associated with depression, suicidal ideation, and suicide attempts. Finally, we expected that the relationship between obesity and suicidal ideation would be significantly mediated by sleep problems, health problems, and dissatisfaction with personal appearance.

## 2. Method

### 2.1. Participants

Participants were 401 female undergraduate students of Mexican heritage from a university in South Texas. Their ages ranged from 18 to 25 years (*M* = 20.2; *SD* = 1.98).

### 2.2. Instrument and Measurement of Variables

The American College Health Association-National College Health Assessment II (NCHA-II) is a national research survey in the United States that collects data on college students’ health behaviors, habits, and perceptions. The data utilized in the present study were from the NCHA-II given to students at a university in South Texas (ACHA, 2017). Ten thousand students from the South Texas university—out of a student enrollment of 27,560—were randomly selected to participate in the NCHA-II. The invitations to participate were sent via email. The final sample consisted of 798 students who reported no missing data.

After giving electronic consent, participants completed the NCHA-II through the Qualtrics online survey platform (Qualtrics, Provo, UT, USA). The analyses for the present study captured all female respondents of Mexican heritage aged 25 and younger.

#### 2.2.1. Suicidal Ideation

In the NCHA-II, participants were asked whether they had seriously considered suicide. Possible responses included: 1 = No, never; 2 = No, not in the last 12 months; 3 = Yes, in the last 12 months; 4 = Yes, in the last 30 days; 5 = Yes, in the last 2 weeks.

#### 2.2.2. Suicide Attempts

Participants were asked whether they had ever attempted suicide. Possible responses included: 1 = No, never; 2 = No, not in the last 12 months; 3 = Yes, in the last 12 months; 4 = Yes, in the last 30 days; 5 = Yes, in the last 2 weeks.

#### 2.2.3. Depression

Participants were asked whether they had felt so depressed that it was difficult to function. Possible responses included: 1 = No, never; 2 = No, not in the last 12 months; 3 = Yes, in the last 12 months; 4 = Yes, in the last 30 days; 5 = Yes, in the last 2 weeks.

#### 2.2.4. Sleep Problems

Sleep problem scores were derived by summing participant responses on insomnia (during the past week, how many days have you had an extremely hard time falling asleep?), sleepiness (how many days have you felt sleepy during the day), and feeling tired during the day (how many days have you felt tired during the day?). The possible overall score for sleep problems ranged from 0 to 21. The coefficient alpha for sleep problems was 0.76, thus indicating acceptable internal consistency among the three items.

#### 2.2.5. Health Problems

Participants were asked whether, within the last 12 months, they had experienced a “personal health issue” that was traumatic or very difficult to handle (0 = No; 1 = Yes). This item was used as a proxy indicator of health problems.

#### 2.2.6. Dissatisfaction with Personal Appearance

Participants were asked whether, within the last 12 months, they had experienced concerns about their “personal appearance” that were traumatic or very difficult to handle (0 = No; 1 = Yes). This item was used as a proxy indicator of dissatisfaction with personal appearance.

#### 2.2.7. Obesity

Body mass index (BMI) was used to estimate obesity and was calculated according to participants’ reported height and weight.

## 3. Results

### 3.1. Descriptives and Correlations

The mean for BMI was 26.7 (*SD* = 6.9); the mean for sleep was 9.26 (*SD* = 4.2); and the mean for depression was 2.44 (*SD* = 1.5). The mean for suicidal ideation was 1.47 (*SD* = 1.02), and the mean for suicide attempts was 1.12 (*SD* = 0.42).

According to BMI data, 4.8% of participants were underweight, 46.5% were of a healthy weight, 21.7% were overweight, and 27% were obese. Twenty-four percent (23.8%) of participants reported lifetime suicidal ideation; 12.3% reported ideation during the past year; 6.3% reported ideation during the past month; and 4.8% reported ideation during the past 2 weeks. Nearly ten percent (9.5%) of participants had attempted suicide; 2% reported a suicide attempt during the past year; and 0.2% reported a suicide attempt during the past 2 weeks.

Sixty-one percent (60.9%) of participants reported feeling depressed at some point in their lives; 39.9% reported feeling depressed during the past year; 26.6% reported feeling depressed during the past 30 days; and 16.8% reported feeling depressed during the past 2 weeks. Finally, during the past year, 25% of participants reported experiencing health problems and 36.1% reported being dissatisfied with their personal appearance.

Table 1 shows the correlations between the variables. As expected, greater BMI was significantly associated with greater sleep problems, health problems, dissatisfaction with personal appearance, depression, suicidal ideation, and suicide attempts. In addition, as expected, greater sleep problems, health problems, and dissatisfaction with appearance were each significantly associated with increased depression, suicidal ideation, and suicide attempts.

### 3.2. Mediation Analysis

Table 2 summarizes the regression findings for our mediation analysis. The table lists the regression paths tested, path coefficients, and *p* values of the coefficients to demonstrate that the regression path conditions necessary for parallel multiple mediation (MacKinnon, 2000; MacKinnon et al., 2002; VanderWeele, 2016) were met.

**Figure 1:**
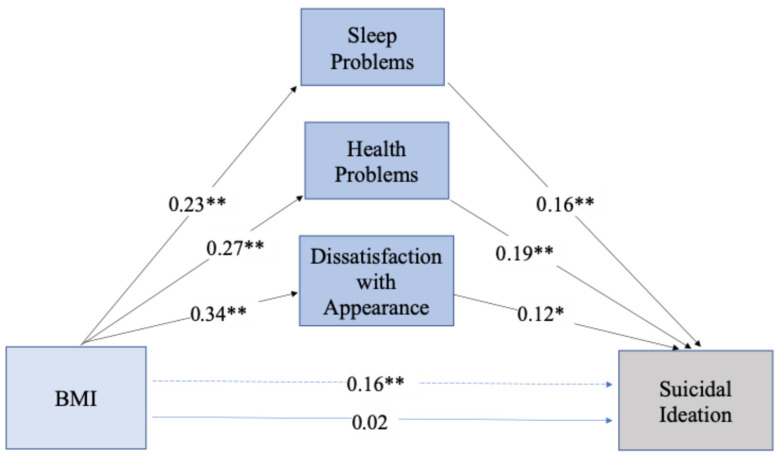
Sleep problems, health problems, and dissatisfaction with personal appearance mediate the relationship between BMI and suicidal ideation. *Note:* * *p* ≤ 0.05 and ** *p* ≤ 0.001.

As indicated by the total effect, BMI was a significant predictor of suicidal ideation. After simultaneously entering sleep problems, health problems, and appearance dissatisfaction into the regression equation, the effect of BMI on suicidal ideation was reduced and became non-significant. In addition, BMI significantly predicted sleep problems, health problems, and appearance dissatisfaction. Sleep problems, health problems, and appearance dissatisfaction were significant predictors of suicidal ideation, while controlling for BMI and the other mediators. Finally, according to Sobel tests, BMI had significant indirect effects on suicidal ideation through sleep problems (*z* = 2.7, *p* = 0.004), health problems (*z* = 3.0, *p* = 0.001), and appearance dissatisfaction (*z* = 2.0, *p* = 0.02), thus suggesting that sleep problems, health problems, and appearance dissatisfaction served as mediators in the relationship between BMI and suicidal ideation and that health problems was the most influential mediator, followed by sleep problems (MacKinnon, 2000). Our overall analysis suggests that the relationship between BMI and suicidal ideation was fully mediated by sleep problems, health problems, and appearance dissatisfaction. Figure 1 depicts the overall parallel multiple mediation model.

## 4. Discussion

Our overall findings suggest that in young Latina adults of Mexican heritage, higher BMI status is associated with an increase in sleep problems, health problems, and personal appearance dissatisfaction, which are associated with greater suicidal ideation. Our study adds to the limited literature on suicide risk in Latina college students as it contributes new risk factors to the limited set of risk factors previously identified by research (Chang et al., 2019; Chesin & Jeglic, 2012).

Moreover, our study contributes new knowledge in response to the question of why obesity and suicide behavior are connected. Previous research has found that self-perception of weight status (Eaton et al., 2005; Minor et al., 2016) and bullying victimization (Van Vuuren et al., 2019) mediated the relationship between obesity and suicidal ideation in female adolescents, and that perceived burdensomeness mediated this relationship in female adults (Dutton et al., 2013). To our knowledge, however, no published research has directly examined sleep problems, health problems, and personal appearance dissatisfaction as mediators in the relationship between obesity and suicidal ideation.

Previous findings provide some verification for the sense of direction in the mediation pathways we proposed. For example, previous research findings indicated that obesity contributes to insomnia, sleep apnea, and excessive daytime sleepiness (Amiri, 2023); that obesity has a clearly measurable impact on various physical health outcomes (Dixon, 2010); and that individuals with obesity are significantly less satisfied with their appearance compared to individuals who are not obese (Weinberger et al., 2016). In addition, sleep problems (Liu et al., 2020), physical health conditions (Ahmedani et al., 2017), and dissatisfaction with personal appearance (Stockton, 2013) are significant predictors of suicide behavior.

Previous research (Altabe, 1998; Lindberg & Stevens, 2011) has found that Latina women are more accepting of their body image and size compared to Anglo women, thus suggesting that factors such as obesity and appearance are not culturally relevant determinants of mental health in Latina women. Our findings strongly suggest, however, that being overweight may indeed lead Latina women to feel dissatisfied with their appearance and that these perceptions are related to negative mental health.

### Research and Clinical Implications

Although, as detailed above, the directionality of variables appears empirically sound, because of the cross-sectional nature of our study, it is not possible to firmly establish the directionality of variables and thus the existence of reciprocal pathways is possible. Prospective research is thus needed to substantiate the mediation and the directionality of variables. Moreover, because we used the NCHA-II, our variables were limited to those included in its database. Some of the variables—health problems and appearance dissatisfaction, in particular—were measured in an abbreviated fashion and may not have fully captured the essence of their constructs. Future research should therefore utilize more comprehensive measures of depression, suicide behavior, and possible mediators. Future research should also explore additional variables that may serve to mediate the relationship between obesity and suicide behavior in young Latina adults of Mexican heritage. These include discrimination, self-efficacy, guilt and stigma associated with weight gain, body shame, and body esteem. Finally, future research should replicate our findings in other Latina groups and generalize our findings to other age and ethnic-cultural groups.

Given that the relationship between BMI and suicidal ideation in our study is largely explained through the influence of our mediators, we recommend that *all* future research on obesity and suicide behavior incorporate possible mediators into their research design to examine *why* obesity and suicide behavior are connected. Not incorporating possible mediators into future research designs might otherwise leave the impression that BMI itself has a strong direct effect on suicide. Moreover, the identification of significant mediators would have important clinical implications as such findings would identify possible pathways for the treatment and prevention of suicide behavior. Our findings, for example, suggest that, for young Latina adults who struggle with weight gain and suicidal thoughts, interventions that strengthen positive perceptions of personal appearance may help to reduce suicide risk. Our findings further suggest that health interventions that reduce obesity and enhance physical health may also help to reduce suicide behavior.

## Figures and Tables

**Table 1:** Correlations Between Variables.

	Sleep Problems	Health Problems	Appearance Dissatisfaction	Depression	Suicidal Ideation	Suicide Attempts
Body Mass Index	0.23 ***	0.35 ***	0.33 ***	0.16 ***	0.16 ***	0.11 **
Sleep Problems	--	0.30 ***	0.31 ***	0.40 ***	0.26 **	0.19 ***
Health Problems	0.30 ***	--	0.47 ***	0.32 ***	0.30 ***	0.23 ***
Appearance Dissatisfaction	0.31 ***	0.47 ***	--	0.36 ***	0.26 ***	0.17 ***
Depression	0.40 ***	0.32 ***	0.36 ***	--	0.49 ***	0.27 ***
Suicidal Ideation	0.26 ***	0.30 ***	0.26 ***	0.49 ***	--	0.51 ***

*Note*: ** *p* ≤ 0.01, *** *p* ≤ 0.001.

**Table 2:** Regression Assessment of Sleep Problems, Health Problems, and Appearance Dissatisfaction as Mediators.

Paths	Path Coefficients	*p*-Values
BMI → Sleep Problems	0.23	<0.001
BMI → Health Problems	0.27	<0.001
BMI → Appearance Dissatisfaction	0.34	<0.001
Sleep Problems → Suicidal Ideation	0.16	0.001
Health Problems → Suicidal Ideation	0.19	<0.001
Appearance Dissatisfaction → Suicidal Ideation	0.12	0.02
BMI →Suicidal Ideation (Total Effect)	0.16	0.001
BMI → Suicidal Ideation (Direct Effect)	0.02	0.37

*Note*: The paths from BMI to health problems and BMI to appearance dissatisfaction were assessed through probit regression; thus, the coefficients listed for these paths are Wald coefficients. All other coefficients are standardized beta coefficients.

## Data Availability

The datasets generated during and/or analyzed during the current study are available from the corresponding author upon reasonable request.
